# Expert consensus for in-hospital neurorehabilitation during the COVID-19 pandemic in low- and middle-income countries

**DOI:** 10.12688/wellcomeopenres.16715.1

**Published:** 2021-05-26

**Authors:** Dorcas B.C. Gandhi, Sureshkumar Kamalakannan, Manigandan Chockalingam, Ivy A. Sebastian, Gerard Urimubenshi, Mohammed Alim, Himani Khatter, Stuti Chakraborty, John M. Solomon

**Affiliations:** 1 College of Physiotherapy and Department of Neurology, Christian Medical College, Ludhiana, Punjab, 141008, India; 2 Public Health Foundation of India, Hyderabad, Telangana, 500033, India; 3 Occupational Therapy, School of Health Sciences, National University of Ireland, Galway, Galway, H91 TK33, Ireland; 4 Department of Neurology, St. Stephen’s Hospital, New Delhi, New Delhi, 110054, India; 5 Department of Physiotherapy, School of Health Sciences, College of Medicine and Health Sciences, University of Rwanda, Kigali, Rwanda; 6 Stroke Clinical Trials Group, Cumming School of Medicine, Calgary, Alberta, T2N 1N4, Canada; 7 Department of Neurology, Christian Medical College and Hospital, Ludhiana, Punjab, 141008, India; 8 Department of Physical Medicine and Rehabilitation, Christian Medical College, Vellore, Vellore, Tamil Nadu, 632002, India; 9 Department of Physiotherapy, Manipal College of Health Professions, Manipal Academy of Higher Education, Manipal, Karnataka, 576104, India; 10 Centre for Comprehensive Stroke Rehabilitation and Research, Manipal Academy of Higher Education, Manipal, Karnataka, 567104, India

**Keywords:** COVID-19, Pandemic, Neurorehabilitation, Guidelines, Consensus, Health Systems

## Abstract

**Background: **People with neurological dysfunction have been significantly affected by the ongoing coronavirus disease 2019 (COVID-19) crisis in receiving adequate and quality rehabilitation services. There are no clear guidelines or recommendations for rehabilitation providers in dealing with patients with neurological dysfunction during a pandemic situation especially in low- and middle-income countries. The objective of this paper was to develop consensus-based expert recommendations for in-hospital based neurorehabilitation during the COVID-19 pandemic for low- and middle-income countries based on available evidence.

**Methods: **A group of experts in neurorehabilitation consisting of neurologists, physiotherapists and occupational therapists were identified for the consensus groups. A scoping review was conducted to identify existing evidence and recommendations for neurorehabilitation during COVID-19. Specific statements with level 2b evidence from studies identified were developed. These statements were circulated to 13 experts for consensus. The statements that received ≥80% agreement were grouped in different themes and the recommendations were developed.

**Results: **75 statements for expert consensus were generated. 72 statements received consensus from 13 experts. These statements were thematically grouped as recommendations for neurorehabilitation service providers, patients, formal and informal caregivers of affected individuals, rehabilitation service organizations, and administrators.

**Conclusions: **The development of this consensus statement is of fundamental significance to neurological rehabilitation service providers and people living with neurological disabilities. It is crucial that governments, health systems, clinicians and stakeholders involved in upholding the standard of neurorehabilitation practice in low- and middle-income countries consider conversion of the consensus statement to minimum standard requirements within the context of the pandemic as well as for the future.

## Introduction

Neurological disorders remain one of the major contributors to death and disability globally ^1^. About 7.1% of the global burden of the diseases are shared by neurological disorders ^2^. Neurological disorders are the leading cause of disability-adjusted life years (DALY) contributing 276 million DALYs and the second leading cause of mortality with about 9 million deaths in 2016 globally ^1^. Neurological disorders such as stroke, headache disorders, epilepsy, dementia, Parkinson’s disease, traumatic brain injury and motor neuron disease amongst others can cause motor, sensory, cognitive, and emotional impairments, leading to disability and poor quality of life among those affected ^3^. The past three decades have seen a considerable rise in the absolute numbers of death and disability due to neurological diseases ^4^. In 2017, the worldwide prevalence (counts in thousands) of years lived with disabilities (YLD) caused by neurological disorders was 3,121,435 (95% CI 2,951,124.5–3,316,268.0) with an increase in YLD (percentage change in counts) by 35.1% (95% CI 31.9–38.1) from 1990 to 2007 and by a further 17.8% (95% CI 15.8–20.2) from 2007 to 2017 ^5^.

Neurorehabilitation is a specialised form of rehabilitation that aims to effectively reduce impairments, improve function, and promote participation in patients with neurological dysfunction ^6^. Evidence supporting the benefits of specialised rehabilitation services for a neurological disability is constantly growing ^6^. However, despite the benefits of specialised rehabilitation services for a neurological disability, inaccessibility, non-availability and lack of affordability of rehabilitation services for persons with disability in general, especially in low- and middle-income countries (LMICs) is a huge barrier ^7^. Lack of resources, limited awareness, ineffective health systems, lack of expertise (199 physiotherapists & <50 occupational therapists per million of the population) ^8^, and low priority for chronic illnesses are some of the reasons for the challenges faced in optimal delivery of rehabilitation services in LMICs ^9^.

In addition to the pre-pandemic challenges, the ongoing coronavirus disease 2019 (COVID-19) pandemic has overwhelmed the effective delivery of healthcare and rehabilitation services globally. PWDs (persons with disabilities) who were previously accessing neurorehabilitation services are unable to access these services because of pandemic restrictions. Most of the institutions offering rehabilitation services have either closed or services have been disrupted ^10^. Travel bans have restricted provision of rehabilitation service in the community/home too ^11^. People experiencing neurological disability are particularly more vulnerable in these contexts because the brain pathologies may impair their level of understanding about the pandemic situation and create more confusion and stress to effectively adhere to the restrictions imposed. This creates a double burden for persons with neurological disabilities to effectively manage their disability during the COVID-19 pandemic and other infectious diseases. The needs and the demand for rehabilitation services to meet the needs of people experiencing neurological disability could substantially increase if the situation is not mitigated ^12^.

In the present circumstances, it is implicit that competent hospital-based rehabilitation services are all the more, an indispensable element of healthcare. Rehabilitation is crucial not only for optimising health outcomes in severe cases of COVID-19 with complicated respiratory involvements but also in facilitating early discharge and reducing the risk of readmission ^13, 14^. In addition, non-COVID-19 infected patients with other ailments continue to require optimal rehabilitation services. Infection with COVID-19 has also manifested various neurological associations affecting both the central and peripheral nervous systems (CNS and PNS, respectively) and could lead to potentially life-long disabling conditions without adequate and timely rehabilitative intervention ^15^.

**Figure 1.**
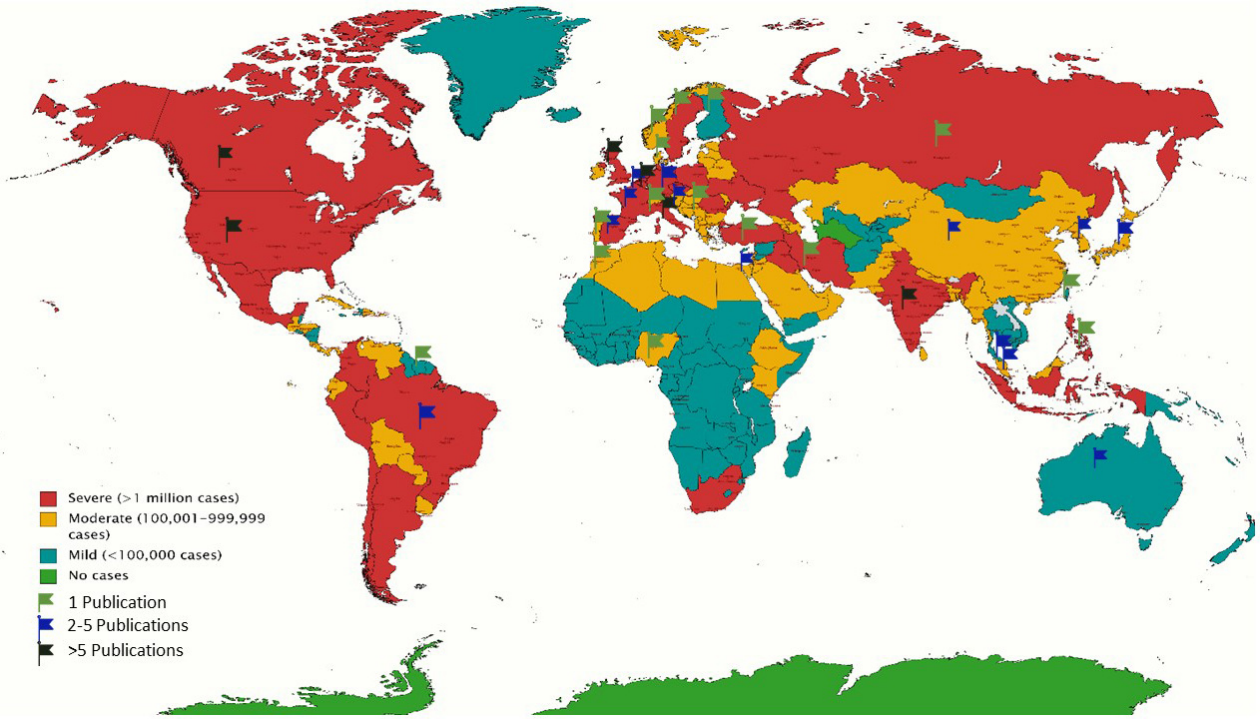
Colour-coded world map depicting coronavirus disease 2019 (COVID-19) global stats as of May 2021. Flags represent the countries with published recommendations for evidence-based neurorehabilitation during the pandemic.

However, the mismatch between demand and resources remains a challenge. For example, the lack or shortage of beds has led to rehabilitation facilities being utilised for other acute patient care; restriction of face-to-face treatment considered to be ‘non-urgent’ has translated into reduced access to vital rehabilitation. Such practices are thereby preventing patients with neurological disorders from regaining lost functional skills ^16^. Safety also remains a concern among rehabilitation professionals due to the need for prolonged and close contact with patients during most neurorehabilitation therapy and from aerosol-generating procedures ^17^. The lack of sufficient evidence-based data on the best practices in rehabilitation that minimize risks from COVID-19 has further impaired the optimal delivery of neurorehabilitation services ^18^. Figure 1 illustrates the incongruity between the global figures of COVID-19 as of May 2021 and the current neurorehabilitation recommendations ^9^. Therefore, there is a need for rethinking the structures and processes for acute in-hospital neuro-rehabilitation ^19, 20^. In this perspective, we aimed to develop the recommendations for in-hospital neurorehabilitation during and after the COVID-19 pandemic which could be a potential basis of reference and guidance for other similar conditions.

The objective of this study was to systematically develop consensus-based expert recommendations for hospital-based neurorehabilitation during the COVID-19 pandemic for low- and middle-income countries based on available evidence.

## Methods

### Study design

**Figure 2.**
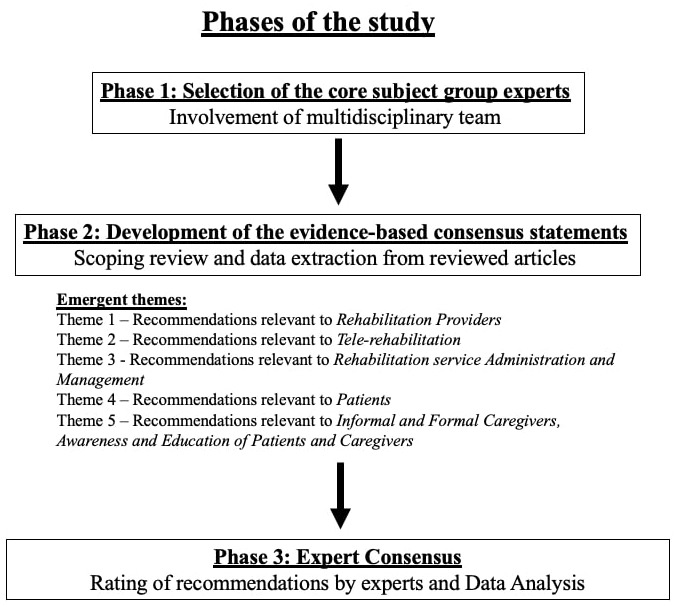
Phases involved in the study.

This study was carried out in India between August 2020 and April 2021 and incorporated a phased approach with a mixed-methods design ( Figure 2). There were three phases including: 1) selection of the core subject group experts, 2) development of the evidence-based consensus statements, and 3) expert consensus. Measures undertaken to address potential sources of bias were as follows:

1) Blinded rating from experts
2) Inclusion of a multidisciplinary expert group to have a comprehensive input

### Ethical approval

Due to the nature of this study, i.e. consensus-based recommendation/guidelines, the authors were informed by the ethics committee (from the lead author’s institution) that ethical approval was not essential. The authors obtained individual written informed consent from each of the experts who were involved in the rating process.

### Phase 1: Selection of the core subject group experts

In this phase, a core group of experts in neurorehabilitation were identified by the lead author and by snowball contacting. The inclusion criteria to have them on board as subject experts and co-authors were as follows: (1) working experience in the field of stroke rehabilitation (2) working experience in or with stroke care/rehabilitation in LMICs having a minimum of ten years. All experts who co-authored this study were approached by the lead author via mail. There was no remuneration provided to any of the authors for their involvement in the study.

The core group had multi-disciplinary expertise in neurorehabilitation and comprised of 1 neurologist, 3 physiotherapists, 3 occupational therapists, a postdoctoral fellow in stroke research, and a statistician. Initial consultation via video-conference on 21 ^st^ August 2020 was held among the core group members to discuss the purpose of the study, and the process for the development of the consensus-based recommendations was finalised. This meeting was conducted to determine the steps to be followed for consensus development. All core members were present, i.e. the 9 authors involved in this study. The first meeting included introductions and development of an overall draft of steps to be followed. The following meetings had specific agendas to assess and decide progress of the work. The core team was also divided into subgroups for each phase of the study and had a leader for each subgroup. The sessions were led by DG.

### Phase 2: Development of the evidence-based consensus statements

A global scoping review was conducted to identify existing evidence and recommendations for neurorehabilitation during COVID-19. Given the extensive resources and processes involved, the detailed scoping review will be published separately. In brief, a six-stage scoping review methodology recommended by the Joanna Briggs Institute was carried out ^21^. The objective of the review was to identify available guidelines, position statements, consensus and recommendations related to neurological rehabilitation during the COVID-19 pandemic globally. This review aimed to explore the existing guidelines for acute neurorehabilitation globally during the context of COVID-19. A comprehensive search strategy was developed using MeSH terms for the concepts related to the aim and the search were run in MEDLINE and CINAHL. Searches were run on 12 ^th^ September 2020. Studies to be included were screened and selected by two independent reviewers (MC and MA). Data were extracted, charted, and collated for expert consensus by four independent reviewers (DG, IS, HK, SC) from the included studies.

From the scoping review, literature related to the objectives were identified. Data related to in-hospital neurorehabilitation for any neurological condition during the COVID-19 pandemic were extracted from the included studies. Only those statements/data that had a level of evidence ≥2b according to the Oxford levels of evidence were synthesised to develop statements for consensus among the expert group ^22^. A list of evidence-based statements for neurorehabilitation during COVID-19 was generated. These statements were converted to recommendations for consensus. The recommendations were thematised and presented under five themes. Coding was done by 3 authors (DG, HK, IS). Themes were identified after the data was extracted to be able to extract as much data as possible that is relevant to the topic. Similar data were then grouped under specific themes. 

Themes identified were:

Theme 1: Recommendations relevant to *Rehabilitation Providers*

Theme 2: Recommendations relevant to *Tele-rehabilitation*

Theme 3: Recommendations relevant to *Rehabilitation service Administration and Management*

Theme 4: Recommendations relevant to *Patients*

Theme 5: Recommendations relevant to *Informal and Formal Caregivers, Awareness and Education of Patients and Caregivers*

Each theme of recommendations was further divided into two sub-themes based on whether the patients tested positive or negative for COVID-19 while receiving neurorehabilitation service in the hospital. The detailed list of all these recommendations is provided as extended data ^23^.

Four statements were further added to the first two themes (three to theme 1 and one to theme 2). There appeared to be specific gaps in the recommendation list, and hence these four statements were exclusively added by the authors. These 75-statement document ^23^ along with the additional four statements formed the basis for expert consensus.

### Phase 3: Expert consensus

A concerted attempt was made to reach out to such experts to partake in this phase of consensus development through the authors’ contacts and snowball sampling strategies. The expert group was created in such a way to include various members of a multidisciplinary team. The experts were chosen if they had experience in the field of neurorehabilitation and had worked in or with LMIC settings. A total of 17 experts were identified and invited to take part via email. The communication to experts included an invitation letter, information leaflet and consent form (see extended data ^23^). There was no remuneration provided to the experts and participation was completely voluntary.

The list of 75 statements that were finalised in phase 2 were emailed to the experts who consented to partake in the study. The participants were requested to rate the relevance of each of the 75 statements on a 5-point Likert scale for relevance to in-hospital neurorehabilitation during COVID-19 (with 1 being least relevant and 5 being most relevant). Missing responses were asked to be completed by the experts until a complete response was obtained for all the statements in the document ^24– 26^. All the responses were entered in Microsoft Excel and the proportion of experts with agreement score of ≥3 was calculated using frequency distribution in SPSS version 26.0.

## Results and discussion

In total, 13 experts consented to participate. The expert group consisted of physiotherapists, occupational therapists, a clinical psychologist, speech & language therapist and nurses with minimum ten years of experience as clinicians, practitioners and researchers, especially in LMICs. Demographic details of the experts (profession, gender) are available as extended data ^23^.

Recommendations receiving a score of ≥3 were considered as strong agreement and thus considered for calculation of the percentage of consensus. Out of these, 72 recommendations received an agreement score of ≥3 by 80% or more of the expert participants ^27^. These statements were compiled as the expert consensus statements for the in-hospital neurorehabilitation in LMICs recommendations in all of the five themes mentioned above. These expert consensus statements are presented in Table 1– Table 5. Recommendations with an expert score of 1 and 2 were considered to demonstrate poor agreement and were excluded.

**Table 1.** Recommendations for ‘Rehabilitation Providers’. COVID-19=coronavirus disease 2019.

IN COVID-19 POSITIVE/SUSPECTS	IN COVID-19 NEGATIVE CASES
Screening/ monitoring: • Checklists for COVID ward nurses to screen and monitor fatigue, anxiety, depression, pulmonary function, sensorimotor function, cognition for necessary referrals and measures to be implemented • Continue with Dysphagia screening within 1st 4 hours for post stroke cases and dysphagia management for those relevant, with adequate PPE to prevent exposure and transmission • Consider new opportunities such as using digital biomarkers in measuring or monitoring the functions remotely without in-person contact • Periodic testing for infection of staff Delivery of treatment: • Rehabilitation to be allowed only with adequate training and implementation of hand hygiene protocols, donning and doffing of PPE and self-quarantine as per local guidelines • Goal setting: Plan for most-relevant and most-needed goals for each patient • Training awareness and measures to avoid or reduce risk of aerosol generating procedures during interventions and activities (like aphasia and dysphagia management, sputum clearance techniques, Chest PT etc.) • Relevant safety & chest/pulmonary rehabilitation measures in patients on immunosuppressive therapies and/or with bulbar/respiratory muscle weakness such as MG or lambert Eaton myasthenic syndrome, who may be at a higher risk of contracting the infection or experiencing severe manifestations of COVID-19 • Maximize effectiveness of each patient encounter by grouping together different components of rehabilitation into a single bedside visit • Avoid group therapy sessions for established COVID-19 cases • Avoid close contact between rehabilitation professionals and COVID-19 positive patients for longer than 10 minutes	• Training and implementation of hand hygiene protocols for health professionals • Bed-side therapies to be provided within patient’s room without need for shifting to common rehabilitation rooms • Group therapy sessions should be taken up only when necessary and during the session, maintain a minimum distance of 2 meters between participants and rehab professionals • PTs, OTs to plan recommend video or telephonic appointments with orthotists or equipment specialist as per need of patients • Regular monitoring of medical vital parameters, body temperature (<37.5°C) and oxygen saturation to detect possible symptomatic patients as soon as possible • Continue physiotherapy and standard in-hospital rehabilitation of stroke patients while using masks and gloves • Early assessment of cognitive health in patients (especially those at high risk i.e. with postcritical care or with residual cognitive impairment) • Periodic testing for infection of staff.

**Table 2.** Recommendations for Tele-rehabilitation. COVID-19=coronavirus disease 2019; SOP=standard operating procedure.

IN COVID-19 POSITIVE/SUSPECTS	IN COVID-19 NEGATIVE CASES
• Virtual evaluation via computers/tablets/smartphones and with the help of nurses posted in COVID ward • Use of tele-consultation to address patient and caregiver concerns and motivation (virtual ward rounds) • Primary aims of rehabilitation should be: relieving symptoms of dyspnea, psychological distress and improving participation in rehabilitation, physical function and quality of life • Measures to improve/avail/approve/educate users (patients and rehabilitation providers) on hardware and software equipment, develop SOPs and process flow charts for video consultations and service delivery • Education in best practice guidelines of telemedicine for use in COVID wards • Systems for neurologically ill patients to speak to and interact with families with facilitated teleconferencing to avoid depression, anxiety or feelings of abandonment during isolation due to COVID-19 • Nurses and/or caregiver (if allowed) should be trained in the designated areas through tele-rehabilitation. • Use of secure virtual care like Zoom, Skype, Facetime for therapy delivery through nurses posted in COVID wards (mobility exercises, Upper extremity training, ADL training and assessment, speech/swallowing, cognitive rehabilitation, respiratory, sensorimotor and psychological therapy interventions) • Promote timely and remote speech language, emotional and social health intervention measures where feasible • Self-administered therapies with the supervision via online/video-demonstration/ written or diagrammatic representations to be used • Use of telemedicine to implement self-management strategies under supervision to reduce stress, increase coping or increase physical exercise in PD patients • Tele-rehabilitation and tele-monitoring methods (use of sensors activated on patient phones or watches, attached to their clothes etc.) are useful for specific symptoms, such as tremor, gait, and falls	• In preparation for discharge: Education in best practice guidelines of telemedicine. • In preparation for discharge: measures to improve/avail/approve/educate users on hardware and software equipment, develop SOPs and process flow charts for video consultations and service delivery • Early discharge is encouraged for patients who can follow a rehabilitation program at home, if their clinical situation permits this. The development of tele-rehabilitation programs should be considered.

**Table 3.** Recommendations for Rehabilitation Administration, Management and Process Flow. COVID-19=coronavirus disease 2019; SOP=standard operating procedure.

IN COVID-19 POSITIVE/SUSPECTS	IN COVID-19 NEGATIVE CASES
• Risk-benefit analysis of each protocol to decide for each effort if it should continue and Removal of non-essential steps in protocols that require in-person interactions • Compulsory up-gradation of contemporary procedural skills and knowledge through online courses for COVID wards with documentation of the same • Designating specific areas/units for rehab of COVID-19 positive patients wherever feasible • Develop criteria to categorize patients into ‘video-visit eligible’ and ‘video visit ineligible’ groups • Hybrid models of care through telemedicine: (1) limited clinicians see patients face-to-face and others see them virtually (2) models wherein the physician visit is scheduled and follow-up visits with the multidisciplinary team occur ad hoc over time (3) asynchronous visits using recorded video for patients without access to internet • The clinical urgency of ongoing physical, occupational, and speech/language therapies should be evaluated on a case-by-case basis, and their suspension or continuation agreed upon by therapists, physicians, and patients. • Patients are at high risk of developing post-intensive care unit syndrome and should be tracked and followed by rehabilitation departments. They will have long-term cognitive, emotional, and functional needs that we as a field are in prime position to treat. Plan for these patients and seek them out for long-term follow-up. • Develop defined and relevant evaluation checklists and core elements of needs and prevention with respect to rehabilitation in most common neurological conditions. • Develop streamlined order sets to minimize patient encounters in suspected or positive COVID-19 to a maximum of 4 patient encounters per 24 hours, while attempting to ensure adequate patient surveillance • Suspension of caregiver visits to hospitalized patients except exceptional cases • Suspension of all rehabilitation activities that require movement between rooms and floors • A parallel reconstruction of commissioning, reducing the emphasis on ‘specialist rehabilitation’ by empowering COVID ward nurses to take up these roles	• In preparation for discharge: Structured and organized implementation of tele- health programs with appropriate SOPs dealing with selecting a telehealth platform, developing a documentation system, Identifying and obtaining necessary resources (personnel and supplies) and identifying recruitment resources and developing recruitment strategies • Compulsory up-gradation of contemporary procedural skills and knowledge of rehabilitation providers through online courses with documentation of the same • In preparation for discharge: directing patients to websites and other resources that are updated regularly is paramount, so that they have up-to-date information when they choose to access the information • Ensuring that every patient with persistent disability is seen by the rehabilitation service from the outset, preferably from first contact with healthcare • Filtering patients before admission to rehabilitation by ensuring multiple negative results on consecutive COVID-19 tests

**Table 4.** Recommendations for patients. COVID-19=coronavirus disease 2019; MET=metabolic equivalent of task.

IN COVID-19 POSITIVE/SUSPECTS	IN COVID-19 NEGATIVE CASES
• Special chest and pulmonary rehabilitation measures for COVID-19 positive cases with Myasthenia Gravis, Lambert Eaton myasthenic syndrome, Motor neuron disease etc. • Patients with COVID-19 who experience the following symptoms: severe sore throat, body aches, shortness of breath, general fatigue, chest pain, cough or fever should avoid exercise (>3 METs or equivalent) for between 2 weeks and 3 weeks after the cessation of those symptoms. Prolonged exhaustive or high intensity training should be avoided • Use of e-diaries for screening development or progression of nonmotor symptoms, such as pain or constipation and Paroxysmal events (eg, migraine, seizures) • Therapy services (when appropriate) should also emphasize teaching safe rehabilitative exercises that can be done by the patient ‘as homework’ when alone. • In case of need for graded increase of verticality with tilt table and other exercises to improve respiratory function for bed-ridden patients, ensure sterile equipment by compulsory sanitization after use for every patient. • Train COVID ward nurses to initiate low intensity exercise (≤3 METs or equivalent) for patients who require oxygen therapy, while concurrently monitoring vital signs (heart rate, pulse oximetry and blood pressure). Gradual increase in exercise as per symptoms supervised by Physiotherapists. • Color-coded and picture-based RPE (Rate of perceived exertion) handouts to patients for better understanding. Adjust tele-exercise regimen according to RPE scoring of patients periodically • Develop alternative measures that correlate well with spirometry but are simpler and carry no increases risk of infection (e.g., counting out loud, vocalizing a sound). • Avoid device-based therapies where equipment/device would have to be shared among patients	• Color-coded and picture-based RPE (Rate of perceived exertion) handouts to patients for better understanding. Adjust tele-exercise regimen according to RPE scoring of patients periodically • Relevant safety & chest/pulmonary rehabilitation measures in patients on immunosuppressive therapies and/or with bulbar/respiratory muscle weakness such as MG or lambert Eaton myasthenic syndrome, who may be at a higher risk of contracting the infection or experiencing severe manifestations of COVID-19, and those with Motor Neuron Diseases who are more prone to bulbar or respiratory muscle weakness and threat of pneumonia from COVID-19 infection

**Table 5.** Recommendations for informal and formal caregivers, awareness and education of patients and caregivers. COVID-19=coronavirus disease 2019.

IN COVID-19 POSITIVE/SUSPECTS	IN COVID-19 NEGATIVE CASES
• Education of patients and family that their interactions with the patient and physicians will be limited to telephone, video conferencing or the like. • Patients to be educated about their condition and strategies for self-recovery. • Training and use of Virtual ancillary services whenever necessary. • Reassurance should be given that milder neurological symptoms like headache, dizziness, loss of smell or taste, and sensory changes are likely to improve with minimal intervention	• Therapy training to the caregivers is essential if they are allowed in the designated areas as per hospital protocols • Education on patient self-management; carers (family and professional) being taught how to support self-management; how to facilitate practice, and/or to provide care safely; carers being encouraged to facilitate social integration • Providing patient/family education for self-care after discharge from inpatient rehabilitation at either acute or subacute settings • Education on continuing rehabilitation care in the outpatient setting, and at home through ongoing therapy either in-person or via telehealth.

To the four additional recommendations, experts were asked to respond with an explanation of their agreement/disagreement. Out of four, 80% or more consensus was received for two recommendations, namely,

(1) Rehabilitation providers (including COVID-19 ward nurses) refer to case history and details from the patient file before the therapy session to reduce the amount of time spent at bedside (92.3% consensus), and
(2) Develop protocols for safe, effective and feasible tele-rehabilitation implementation during COVID-19 (84.6% consensus).

However, it was emphasised that such protocols should allow for therapy dosages to be customizable according to patient needs, approved by rehabilitation professionals and considerate of the safety and privacy issues of both rehabilitation providers and patients. Experts also suggested that in-person hands-on therapy should be initiated once a patient is tested negative for COVID-19.

Expert consensus for key aspects of in-hospital neurological rehabilitation services was specific to the rehabilitation service providers, patients, formal and informal caregivers of affected individuals, rehabilitation service organization, and administrators. The consensus statements were also classified according to the levels of evidence. There were specific components that were considered important by the experts in each of these key aspects. For service providers, it was training, implementation, appropriate use of Personal Protective Equipment, adequate safety measures, prioritized therapeutic goal setting, patient safety and therapy effectiveness. For patients, this was related to comprehending symptoms of COVID-19, therapeutic exercises regime including intensity, use of assistive devices or equipment for therapeutic exercise, postural stabilisation and documentation of practice. To our knowledge, ours is the only consensus-based guideline developed to date, addressing the aspect of in-hospital based neurorehabilitation during the ongoing pandemic, and its transferability and application to other similar airborne outbreaks. Previous consensus guidelines have either addressed acute management of stroke in LMICs, neurorehabilitation in LMICs, not specific to in-hospital setting or post-COVID rehabilitation as a whole ^28– 30^.

The consensus statements for caregivers of hospitalised individuals were related to education, training, use of tele-rehabilitation services and reassurance. The consensus statements for management were related to the use of hybrid models of care, organization of strategic pathways for care and rehabilitation, developing criteria as well as prioritization of patient safety and need-based therapeutic engagement with or without caregiver engagement. Lastly, for tele-rehabilitation, the consensus was predominantly related to developing and implementing of secure tele-consultation and tele-rehabilitation services for patients with neurological disability and educating the users and rehabilitation service providers about tele-rehabilitation.

These aspects have to be considered highly crucial and essential during the provision of in-patient neurological rehabilitation services for patients affected by neurological disability who may or may not be tested positive for COVID-19 in the pandemic situation. Although the pandemic seems to be settling down globally, these consensus statements might prove useful during the subsequent waves of the pandemic and also in the post-pandemic future.

The consensus statements need to be contextualised according to the settings. Though the consensus statements came from experts from and with experience working in low-resource settings, it may be useful in all the settings irrespective of the availability of resources. However, implementation of these statements requires contextualisation, especially with respect to resource availability. Highly developed health care systems with adequate resources might have to prioritize rigour in implementation, whereas low resource settings with poor health systems must prioritize relevance. Knowledge, skills and competencies of the rehabilitation professionals in infection control, personal safety and tele-rehabilitation needs to be tested and trained to ensure the appropriate delivery of the recommendations. Frequent faculty development programs could be organised to ensure capacity building and quality delivery of service. 

This study does have its strength and limitations. Firstly, expert consultations, focus group discussions and consensus meetings were conducted virtually as opposed to the in-person meetings, given the pandemic situation. The number of experts chosen for the consensus were representative of a limited geographical area. Both these limitations are considered to have reduced the number of recommendations. However, the expertise and experience of the expert group was diverse and hence it is expected that this would have not compromised the comprehensiveness and overall representation for the consensus. This study is one of the first to develop an in-hospital neurorehabilitation consensus during the COVID-19 pandemic. The expert recommendation was developed through a methodologically rigorous process (a systematic scoping review). The mix of methods for development of the recommendation and the Delphi process to arrive at consensus ensured that the recommendation statements were evidence-based, substantiated by expert consensus. This enhances generalizability and pragmatic implementation in clinical practice.

## Conclusion

Given the current experiences of combating the pandemic worldwide, there is paucity of evidence and guidelines for ensuring patient safety and effective rehabilitation service provision for neurologically disabled patients admitted in the hospitals with or without COVID-19. This consensus statement envisages to provide key recommendations that can be optimised to enhance patient safety and service effectiveness. Systematic implementation of the consensus statement is of utmost importance to empower neurological rehabilitation service providers and patients with neurological disability. It is crucial that governments and health systems in low- and middle-income countries consider inclusive planning and policy making to convert the consensus statements to minimum standards for neurorehabilitation practice in this pandemic context and in the future.

## Data availability

### Underlying data

Open Science Framework: Expert Consensus for in-hospital neurorehabilitation during the COVID-19 pandemic in low-and-middle income countries. https://doi.org/10.17605/OSF.IO/HCSX7 ^27^.

This project contains the following underlying data:

- Consensus paper rating_Raw_Data.xlsx

### Extended data

Open Science Framework: Expert Consensus for in-hospital neurorehabilitation during the COVID-19 pandemic in low-and-middle income countries. https://doi.org/10.17605/OSF.IO/39MF4 ^23^.

This project contains the following underlying data:

- Supplementary File.docx (The information leaflet consisting of instructions as well as elaborate list of 75 statement recommendations which was sent out to the experts)
- Invitation and Instructions for Experts.pdf

Data are available under the terms of the Creative Commons Attribution 4.0 International license (CC-BY 4.0).
