# Supplementary material for: Expert consensus for in-hospital neurorehabilitation during the COVID-19 pandemic in low- and middle-income countries
Source: Wellcome Open Res. 2021 May 26;6:130. [Version 1] doi: 10.12688/wellcomeopenres.16715.1 (PMC8787554; doi:10.12688/wellcomeopenres.16715.1)
Supplement: Peer review history

# Peer review: Reviewer response for version 1

**DOI:** 10.21956/wellcomeopenres.18432.r46830

**Published:** 2022-01-24

**Authors:** Thomas Platz

**Affiliations:** 1 Neurohabilitation Research Group, Universitätsmedizin Greifswald, Greifswald, Germany; 2 Institute for Neurorehabilitation and Evidence-based Practice ("An-Institut"), BDH-Klinik Greifswald, University of Greifswald, Greifswald, Germany

The paper had been submitted as Research Article and reports about the methods and results of a multidisciplinary expert consensus project for in-hospital neurorehabilitation during the COVID-19 pandemic and the healthcare context of low- and middle-income countries. It addresses aspects that are considered relevant for rehabilitation providers, administration and management, patients, and caregivers both for hospital-based neurorehabilitation of COVID-19 cases and non-COVID-19 cases in need for neurorehabilitation during the pandemic. The consensus project aimed to provide and agree on practice recommendations relevant for these stakeholders during the pandemic in low- and middle-income countries. Such guidance is important and can serve these stakeholders as orientation for healthcare provision and the development of regionally contextualized clinical pathways.

 Some comments might be given that could help to further improve the manuscript.

***Author and expert review group:***

 The authors mention that the experts chosen for the consensus were representative of a limited geographical area; this might not only have reduced the number of recommendations, but might to some extent also affect their global applicability.

 (Neuro-)Psychological problems (emotional and cognitive) are frequent sequelae of COVID-19. Psychology as a discipline could be considered as less strongly represented in the work as might have been ideal (i.e. a limitation).

 In addition, patient representatives seemed not to be involved.

***Healthcare question to be addressed:***

 Recommendations for Neurorehabilitation of COVID-19 cases is strongly related to the specific Neuro-COVID presentations (type of neurological conditions/impairments) seen, their severity, frequency, and any “clusters” of presentation, e.g. Long-/Post-COVID-19. Such information (while available as research data, even as meta-analytic data) is lacking in the manuscript and could be added indicating the major clinical problems faced and hence to be addressed by practice recommendations.

***Methodological issues:***

**Scope of the review undertaken:**

 A scoping review - as conducted in this project - can include any and all types of literature (e.g., primary research studies, systematic reviews, meta-analyses, letters, guidelines, websites, blogs).

 To some extent the manuscript remained unclear what the basis of data extraction for the scoping review was. And, there is uncertainty about any evidence on neurorehabilitation of COVID-19 cases and non-COVID-19 cases in need of neurorehabilitation during the pandemic that was searched for and used (“A scoping review was conducted to identify existing evidence and recommendations”). The authors further state “The objective of the review was to identify available guidelines, position statements, consensus and recommendations related to neurological rehabilitation during the COVID-19 pandemic globally. This review aimed to explore the existing guidelines for acute neurorehabilitation globally during the context of COVID-19.” Explicit inclusion and exclusion criteria for entries and a complete search strategy for at least one major database could be included in the manuscript to clarify the matter.

**Entries retrieved:**

 If the entries searched for had been guidelines, they might have been missed partially due to any non-publication in peer-review journals, but rather online publication by governmental or medical society websites. E.g., in Germany a guideline with consensus-based expert recommendations for in-hospital based rehabilitation during the COVID-19 pandemic (including neurorehabilitation) with 64 recommendations had been published ( https://www.awmf.org/leitlinien/detail/ll/080-008.html, version 2; short publication of version 1 at https://www.aerzteblatt.de/archiv/218662/AWMF-Leitlinie-Rehabilitation-nach-einer-COVID-19-Erkrankung). Such restrictions of the scope of review might be mentioned.

**Type of recommendation developed:**

 It is stated: “From the scoping review, literature related to the objectives were identified. Data related to in-hospital Neurorehabilitation for any neurological condition during the COVID-19 pandemic were extracted from the included studies. Only those statements/data that had a level of evidence ≥2b according to the Oxford levels of evidence were synthesised to develop statements for consensus among the expert group”. The authors correctly note “The objective of this paper was to develop consensus-based expert recommendations for in-hospital based neurorehabilitation during the COVID-19 pandemic for low- and middle-income countries based on available evidence.” Then, however, they state “Phase 2: Development of the evidence-based consensus statements”. As far as can be deduced from the manuscript in its current form, consensus-based expert recommendations were developed, but not evidence-based consensus statements.

 For the development of evidence-based recommendations - with the scope of recommendations aimed for as stated above - the evidence from clinical research (i.e., evidence on hospital-based neurorehabilitation of COVID-19 cases and non-COVID-19 cases in need of neurorehabilitation during the pandemic with a focus on both rehabilitation provision, administration and management, as well as patient and carer information/education) would have systematically been search for, critically appraised, and then practice recommendations would have been deduced within a evidence-to-decision framework (Platz and Owolabi, 2021 ^1^; Platz, 2021 ^2^). If not done, it might be more correct in the given context to speak of “consensus-based expert recommendations”.

**Relevance / agreement/ consensus:**

 “The participants were requested to rate the relevance of each of the 75 statements on a 5-point Likert scale for relevance to in-hospital neurorehabilitation during COVID-19 (with 1 being least relevant and 5 being most relevant).”

 “Recommendations receiving a score of ≥3 were considered as strong agreement and thus considered for calculation of the percentage of consensus.”

 The two constructs, i.e. “relevance” and “agreement” seem to be “mixed-up” here. E.g., the panellists might have had a high degree of agreement that a recommendation was of little relevance, and conversely a low agreement that another was of high relevance. Accordingly, it should be made clear in the manuscript, what the criteria for agreement (and degree of agreement) and methods to analyse agreement were, and how the recommendation selection process was defined a priori considering both factors “relevance” and “agreement” (e.g., something like ‘only recommendations that were considered relevant, i.e. receiving a score of ≥3 out of 5 by a vast majority of experts, i.e. ≥80% were considered to be retained’).

Is the work clearly and accurately presented and does it cite the current literature?

Partly

If applicable, is the statistical analysis and its interpretation appropriate?

Partly

Are all the source data underlying the results available to ensure full reproducibility?

No

Is the study design appropriate and is the work technically sound?

Yes

Are the conclusions drawn adequately supported by the results?

Yes

Are sufficient details of methods and analysis provided to allow replication by others?

Partly

Reviewer Expertise:

Neurorehabilitation, practice recommendation/guideline development

I confirm that I have read this submission and believe that I have an appropriate level of expertise to confirm that it is of an acceptable scientific standard, however I have significant reservations, as outlined above.

## References

[1] : Clinical Pathways in Stroke Rehabilitation: Background, Scope, and Methods.2021; 10.1007/978-3-030-58505-1_2 15-34 10.1007/978-3-030-58505-1_2 36315694

[2] : Methods for the Development of Healthcare Practice Recommendations Using Systematic Reviews and Meta-Analyses. *Front Neurol* .2021;12: 10.3389/fneur.2021.699968 699968 10.3389/fneur.2021.699968 34305801PMC8297739

# Peer review: Reviewer response for version 1

**DOI:** 10.21956/wellcomeopenres.18432.r46833

**Published:** 2021-11-08

**Authors:** Farooq Azam Rathore

**Affiliations:** 1 Armed Forces Institute of Rehabilitation Medicine (AFIRM), Rawalpindi, Pakistan

It is an important manuscript and a useful contribution to the global literature on the role of rehabilitation in COVID-19. This is based on an expert consensus of selected rehabilitation professionals mainly working in India (an LMIC) who gathered virtually to provide consensus recommendations on for in-hospital neurorehabilitation during the COVID-19 pandemic in low- and middle-income countries. 

 My concern is that many guidelines and recommendations have been published for Post COVID Rehabilitation. Although many of them are not specific to LIC/LMIC, it is important to cite them in order to provide the context to the global efforts being done by rehabilitation professionals in different parts of the world.

 Some notable examples are as follows:

https://www.mdpi.com/2077-0383/10/8/1691 ^1^

https://www.europeanreview.org/article/24211 ^2^

https:/www.medicaljournals.se/jrm/content/abstract/10.2340/16501977-2776 ^3^

 Neurorehabilitation is a multidisciplinary team effort with a physiatrist/ Rehabilitation Medicine Physician as an integral and often the team leader. This is the global norm in the majority of the countries where neurorehabilitation services are well established. This consensus panel of 13 experts did not have even a single Rehabilitation Medicine Physician. This is a major limitation of the manuscript which must be mentioned and explained.

 The virtual meeting has been mentioned as one of the weaknesses of this manuscript. I tend to disagree. In fact, this is a strength of this manuscript that a diverse group of experts from different parts of the world was brought together virtually to share their expertise and give recommendations. Please amend.

 The literature search needs to be redone and additional relevant references based on the data and experience sharing from other LIC/LMIC need to be integrated. 

 Other minor comments are as follows:

- Keywords ideally should not be the same words used in the title.
- Consult the MeSH database to choose appropriate keywords. https://www.ncbi.nlm.nih.gov/mesh

Is the work clearly and accurately presented and does it cite the current literature?

Partly

If applicable, is the statistical analysis and its interpretation appropriate?

Not applicable

Are all the source data underlying the results available to ensure full reproducibility?

Yes

Is the study design appropriate and is the work technically sound?

Yes

Are the conclusions drawn adequately supported by the results?

Yes

Are sufficient details of methods and analysis provided to allow replication by others?

Yes

Reviewer Expertise:

Neurorehabilitation, Stroke rehabilitation, Spinal Cord Injury Rehabilitation, Pain Management

I confirm that I have read this submission and believe that I have an appropriate level of expertise to confirm that it is of an acceptable scientific standard, however I have significant reservations, as outlined above.

## References

[1] : A COVID-19 Rehabilitation Prospective Surveillance Model for Use by Physiotherapists. *J Clin Med* .2021;10(8) : 10.3390/jcm10081691 10.3390/jcm10081691 33920035PMC8071011

[2] : Aerobic exercises recommendations and specifications for patients with COVID-19: a systematic review. *Eur Rev Med Pharmacol Sci* .24(24) : 10.26355/eurrev_202012_24211 13049-13055 10.26355/eurrev_202012_24211 33378057

[3] : Rehabilitation setting during and after Covid-19: An overview on recommendations. *J Rehabil Med* .2021;53(1) : 10.2340/16501977-2776 jrm00141 10.2340/16501977-2776 33284353PMC8772378

# Peer review: Reviewer response for version 1

**DOI:** 10.21956/wellcomeopenres.18432.r45254

**Published:** 2021-08-17

**Authors:** Rachel Stockley

**Affiliations:** 1 Stroke Research Team, School of Nursing, Faculty of Health and Wellbeing, University of Central Lancashire, Preston, UK

This is well constructed, interesting and useful study. It is novel and adds to the understanding and practice of stroke rehabilitation in LMICs.

 It is largely well written and clear. There are some minor errors in writing style e.g. "Themes were identified after the data was extracted to be able to extract as much data as possible that is relevant to the topic." which require rewording to increase clarity. It would also be useful to know what qualitative methodology was adopted in the thematic analysis (was it inductive or deductive) and some consideration of the researcher's potential influence on these themes.

 A further unacknowledged limitation is that the recommendations for patients and carers were developed without input from carers or patients. This is important to acknowledge as they may have prioritised/agreed differently on the themes that pertained to them than the healthcare providers.

 Overall, this article adds to an understanding of practice during the pandemic and its authors should be commended on producing it during such a challenging time.

Is the work clearly and accurately presented and does it cite the current literature?

Yes

If applicable, is the statistical analysis and its interpretation appropriate?

Not applicable

Are all the source data underlying the results available to ensure full reproducibility?

Yes

Is the study design appropriate and is the work technically sound?

Yes

Are the conclusions drawn adequately supported by the results?

Yes

Are sufficient details of methods and analysis provided to allow replication by others?

Yes

Reviewer Expertise:

Neurological Rehabilitation, clinical research in stroke.

I confirm that I have read this submission and believe that I have an appropriate level of expertise to confirm that it is of an acceptable scientific standard.
